# Hyperpolarized [1,4-^13^C]fumarate imaging detects microvascular complications and hypoxia mediated cell death in diabetic nephropathy

**DOI:** 10.1038/s41598-020-66265-6

**Published:** 2020-06-15

**Authors:** Christoffer Laustsen, Per Mose Nielsen, Haiyun Qi, Mette Hadberg Løbner, Johan Palmfeldt, Lotte Bonde Bertelsen

**Affiliations:** 1 MR Research Centre, Department of Clinical Medicine, Aarhus University, Aarhus, Denmark; 2 Research Unit for Molecular Medicine, Department of Clinical Medicine, Aarhus University, Aarhus, Denmark

**Keywords:** Kidney diseases, Biomarkers, Preclinical research

## Abstract

Today, there is a general lack of prognostic biomarkers for development of renal disease and in particular diabetic nephropathy. Increased glycolytic activity, lactate accumulation and altered mitochondrial oxygen utilization are hallmarks of diabetic kidney disease. Fumarate hydratase activity has been linked to mitochondrial dysfunction as well as activation of the hypoxia inducible factor, induction of apoptosis and necrosis. Here, we investigate fumarate hydratase activity in biofluids in combination with the molecular imaging probe, hyperpolarized [1,4-^13^C_2_]fumarate, to identify the early changes associated with hemodynamics and cell death in a streptozotocin rat model of type 1 diabetes. We found a significantly altered hemodynamic signature of [1,4-^13^C_2_]fumarate in the diabetic kidneys as well as an systemic increased metabolic conversion of fumarate-to-malate, indicative of increased cell death associated with progression of diabetes, while little to no renal specific conversion was observed. This suggest apoptosis as the main cause of cell death in the diabetic kidney. This is likely resulting from an increased reactive oxygen species production following uncoupling of the electron transport chain at complex II. The mechanism coupling the enzyme leakage and apoptotic phenotype is hypoxia inducible factor independent and seemingly functions as a protective mechanism in the kidney cells.

## Introduction

The worldwide increase in prevalence of diabetes mellitus, is a major risk factor for chronic kidney disease (CKD) which can lead to end stage renal disease (ESRD). Both CKD and ESRD are a growing global public health problem^1–3^. Intra-renal tissue hypoxia has been identified as a common link between several renal pathological conditions^4^ and as such represents an appealing target for diagnostic biomarkers in renal disease.

Recent advances in magnetic resonance imaging (MRI) has introduced the novel hyperpolarized metabolic imaging technology, denoted dissolution dynamic nuclear polarization (d-DNP), to renal application in murine and porcine models^5–11^. This technology allows non-invasive imaging of renal hemodynamics, uptake and conversion of endogenous metabolites in real time^12^. Of particular interest is hyperpolarized [1-^13^C]pyruvate, whose metabolic fate can be traced via the hemodynamic distribution, metabolic uptake and subsequent conversion to either [1-^13^C]lactate, [1-^13^C]alanine or ^13^CO_2_ /H^13^CO_3_ in the human body within seconds after intravenous injection^13,14^.

Hyperpolarized [1-^13^C]pyruvate has been shown to provide accurate metabolic staging of the hypoxic nature associated with several renal complications^11,15–20^, where an increased lactate production both with and without a decreased oxidative phosphorylation (decreased ^13^CO_2_ /H^13^CO_3_ production) is seen. However, in order to determin the underlying mechanism additional biomarkers are needed.

**Figure 1:**
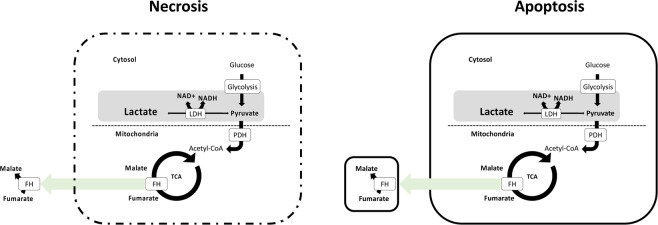
Necrosis is associated with increased cell volume, loss of plasma membrance integrity and leakage of cellular content directly leading to an increased metabolic conversion via free enzymes such as FH. Apoptosis on the other hand is typically chartarized by cell shrinkage, plasma membrane blebbing and the formation of apoptotic bodies.

Hyperpolarized [1,4-^13^C_2_]fumarate measurements have been utilized to pinpoint acute kidney injury (AKI) as seen by an increased primary necrosis, with conversion of [1,4-^13^C_2_]fumarate to [1,4-^13^C_2_]malate via Fumarate hydratase (FH)^8,21^. As [1,4-^13^C_2_]fumarate does not pass the cell membrane under normal conditions (on the time scale of the experiment), this conversion is almost exclusively originating from free FH enzymes lost to the surroundings during cell death (Fig. 1). It is therefore likely that combination of [1-^13^C]pyryvate and [1,4-^13^C]fumarate will allow better discrimination of the underlying pahtphysiology if [1,4-^13^C]fumarate to [1,4-^13^C]malate conversion is absent in chronic kidney disease such as diabetic nephropathy (DN).

Several reports have demonstrated a correlation between urinary fumarate concentration or FH activity and the severity of renal damage in CKD or acute kidney injury (AKI)^8,22,23^. However, the action is less clear in diabetes with opposing results^24,25^.

DN is generally considered to be associated with apoptosis and not necrosis as well as being closely linked to alterations in oxygen metabolism. Here, we investigate the underlying mechanisms associated with cell death and the link to alterations in the oxygen metabolism in the diabetic kidney using [1,4-^13^C_2_]fumarate, combined with plasma and urine biochemical analyses and quantitative cellular proteomics. We test the hypothesis that hyperpolarized [1,4-^13^C]fumarate conversion is largely absent in DN and the apotosis is directly linked to the oxygen metabolic phenotype seen in diabetes.

## Methods

### Animals

**Figure 2:**
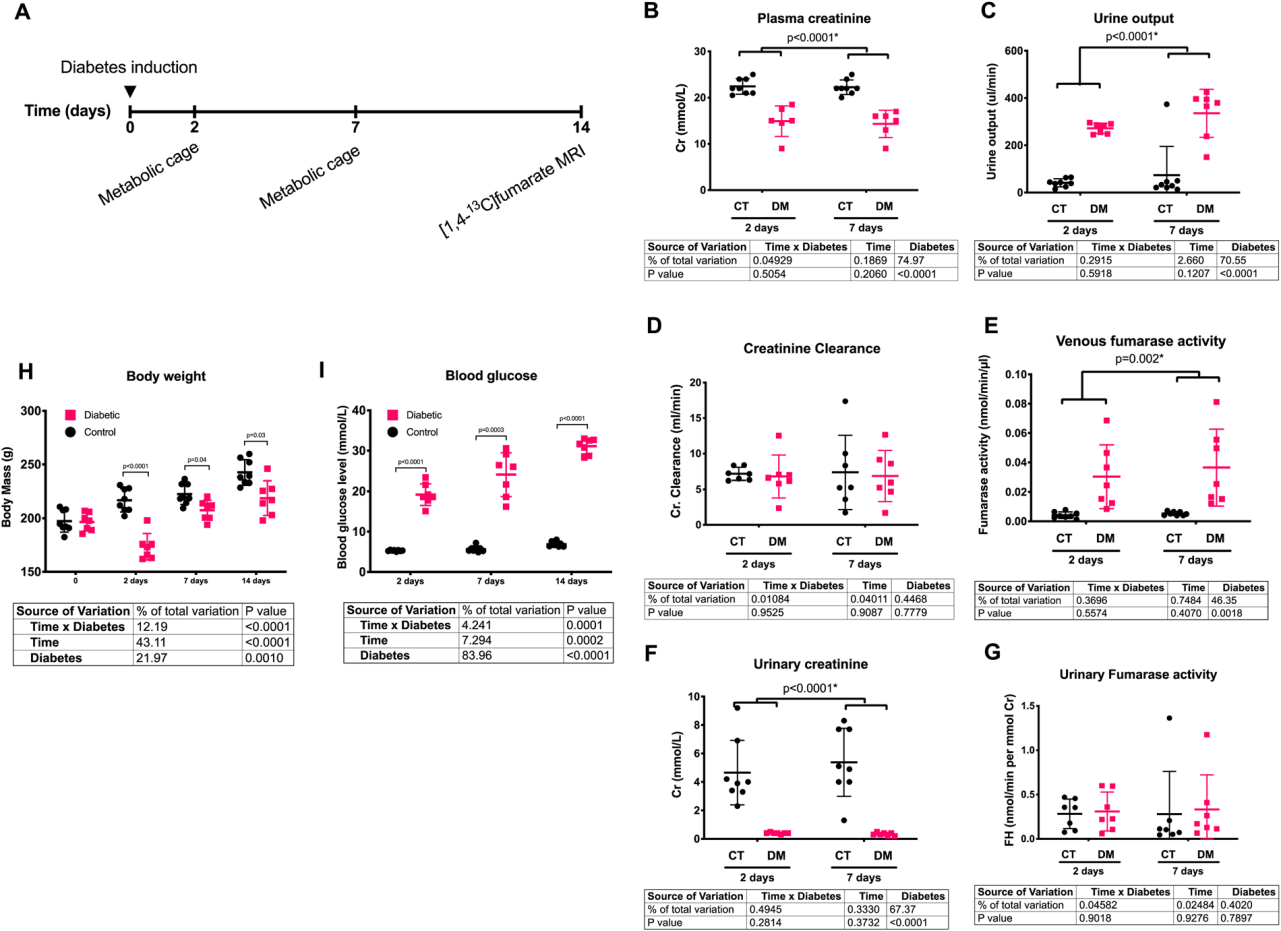
(**A**) Illustration of the experimental setup consisting of two metabolic cage experiments and a final MR exam using hyperpolarized [1,4-^13^C_2_]fumarate. (**B**) Diabetic animals had a clear reduction in plasma creatinine (mmol/L) already two days after indication of diabetes. (**C**) A similar clear effect of diabetes was seen in the urine output (uL/min/kg). (**D)** The creatinine clearance (ml/min/kg) was similar between diabetic and control rats at both 2 and 7 days following induction of diabetes. (**E**) Diabetes was associated with an early fumarate enzyme leakage, observed with a significantly increased plasma FH activity (nmol/min/uL) at both 2 and 7 days after induction of diabetes. (**F**) A significant lower renal function (urinary creatinine level, mmol/L) was observed in diabetic animals already 2 days after induction of diabetes and consistent after 7 days of diabetes. (**G**) Seven days of diabetes was not sufficient to promote urinary protein leakage. No difference were found in the in urinary FH activity corrected for renal function (normalized by the urinary creatinine) between healthy and diabetic animals. (**H**) Diabetic animals had a consistent significantly lower body weight already two days after induction of diabetes. (**I**) Diabetic animals was highly hyperglycemic already at day two after induction of diabetes and throughout the 14 day experimental period. ANOVA summary tables are placed below the individual subfigures.

Sixteen eight-week-old female Wistar rats were included in this study. The rats were kept in cages with a 12:12-h light dark cycle, a temperature of 21 ± 2 °C and a humidity of 55 ± 5%. The rats were randomly grouped in a diabetic untreated group (N = 7, one diabetic rat was sacrificed prior to completion due to animal welfare concerns and thus excluded from the study) and a healthy control group (N = 8). Diabetes was induced by an intravenous injection of freshly prepared streptozotocin (STZ; 55 mg/kg body weight; Sigma-Aldrich, Brondby, Denmark) dissolved in 10 mmol/L cold citrate buffer (pH 4.5). Blood glucose was measured in tail-capillary blood with a Contour blood glucose meter (Bayer Diabetes Care, Copenhagen, Denmark)^11,15,16,20^. Rats were considered diabetic when the blood glucose levels exceeded 15 mmol/L at 48 h after injection of STZ. The animals were placed in metabolic cages for 24 hours at 2 and 7 days after induction of diabetes in order to measure renal functional parameters (Fig. 2A). At day 14, the animals were scanned and subsequently sacrificed. Shortly before the animals were sacrificed, blood from the aorta, urine from the bladder and kidney cortex tissue were sampled acutely. The study was carried out in accordance with Danish National Guidelines for animal care, and was approved by the Danish Animal Experiments Inspectorate under the Danish Veterinary and Food Administration (License no. 2014-15-0201-00327). Fourteen days of diabetes leads to an early diabetic nephropathy model as previously described, for consistency only female rats was used^11,15,16,20,26^.

### Cell culture

The rat kidney epithelial cell line NRK-52E was obtained from Sigma-Aldrich (Sigma-Aldrich, Brondby, Denmark). NRK-52E cells were cultured in Dulbeccos modified eagles medium (DMEM) with low glucose 1000 mg/L (Sigma-Aldrich, Brondby, Denmark) supplemented with 10% fetal bovine serum (Sigma-Aldrich, Brondby, Denmark), 10 units/ml penicillin and 10 mg/ml streptomycin and were maintained in continuous culture at 37°C in a humidified atmosphere (5% CO_2_) in an incubator. Cell number and viability was determined using a trypan blue exclusion test^27,28^. Growth medium was changed every second or third day, and cells were sub-cultured until further measurements at 80% colony confluency. For cellular integrity and sensitivity experiments, two groups of cells were grown with or without high glucose (25 mM) in the culturing medium. Cells were exposed to high glucose for 48 hours to promote a hyperglycemic phenotype^29,30^. In each of the two groups of cells, subgroups were analyzed under the conditions of no-treatment, treatment with 15 μM etoposide (Etoposide, 25 mg; Sigma-Aldrich, Brondby, Denmark) for 24 hours to promote mild cell death dominated by apoptosis, as well as exposing the cells to 3 times of freeze and thaw cycles to promote cell death similar to necrosis^31^. The necrosis serves as a positive control to the apoptotic cell death similar to previous reports^31^.

### Activity assays in plasma, urine, whole renal cortex tissue and mitochondrial fractions

FH activity was measured in plasma, urine, whole renal cortex tissue, and mitochondrial fractions according to the manufacturer’s instructions (Sigma-Aldrich, Brondby, Denmark)^18^. FH activity in the mitochondria and tissue was normalized to the amount of protein in the sample. Plasma and urine FH activity was normalized to the amount of sample added to the assay. The mitochondrial fraction was isolated using Dounce homogenization of freshly dissected renal tissue followed by several centrifugal steps. Mitochondrial purity was verified by Western blotting. The tissue and mitochondrial fractions were then homogenized in FH assay buffer. Analysis was performed in 96-well Costar half plates using a PHERAstar FS microplate reader (BMG Labtech, Birkerod, Denmark). Urine and plasma samples were distributed without pre-treatment in 96-well costar half plates. FH activity in urine was assessed at a wavelength of 670 nm instead of the usual 650 nm because of the presence of background interference^18^.

### Activity and concentrations assays in cell and supernatant fractions

Lactate dehydrogenase (LDH), FH and succinate dehydrogenase (SDH) activity assays as well as fumarate and succinate concentration assays were performed according to the manufacturer’s instructions with few alterations (Sigma‐Aldrich, Brondby, Denmark)^20^. 1 × 10^6^ cells were lysed in mammalian protein extraction reagent M-PER (Fisher Scientific, Hampton, New Hampton, USA) supplemented with a proteinase inhibitor cocktail (Complete, Mini, EDTA-free, Sigma‐Aldrich, Brondby, Denmark) at room temperature (RT) for 15 min before centrifugation and harvest of the supernatant. Analysis was performed in 384 well-plates in a BioTEK synergy plate reader (AH diagnostics, Aarhus, Denmark)^20^. Activity measurements were normalized to protein amount in the sample solution. Protein quantification was measured utilizing a Qubit 3.0 flourometer (Fisher Scientific, Slangerup, Denmark).

### RNA extraction and quantitative PCR

Total RNA was isolated from the renal cortex using a NucleoSpin RNA II mini kit according to the manufacturer’s instructions (AH diagnostics, Aarhus, Denmark)^18^. RNA was quantified by spectrophotometry and stored at −80 °C. cDNA synthesis was performed with a RevertAid First strand cDNA synthesis kit (MBI Fermentas, Burlington, Canada). qPCR was performed using Maxima SYBR Green qPCR Master Mix according to the manufacturer’s instructions (AH diagnostics, Aarhus, Denmark). Briefly, 100 ng of cDNA was used as a template for PCR amplification^18^. The specificity of products was confirmed by melting curve analysis and gel electrophoresis^18^.

### Hyperpolarized experiments

The animals were anesthetized with sevoflurane (2.5% sevoflurane and 2 L/min air) and maintained anesthetized throughout the experiment. Prior to placement in the clinical 3 T MRI scanner equipped with a dual tuned ^13^C/^1^H volume rat coil (GE Healthcare, Waukesha, US) tail vein catheterization was performed for hyperpolarized [1,4-^13^C_2_]fumarate administration. Rectal temperature, pO_2_ and respiration were monitored throughout the MRI session. Each animal was injected with 1.5 mL of hyperpolarized 30 mM [1,4-^13^C_2_]fumarate. The [1,4-^13^C_2_]fumarate was prepared and polarized in a SPINlab (GE Healthcare, Brondby, Denmark) as previously described in^8^.

Hyperpolarized metabolic imaging was performed with a slice-selective ^13^C IDEAL spiral sequence, with a 2 second temporal resolution, initiated at the start of injection, similar to previous reports^8^.

**Figure 3:**
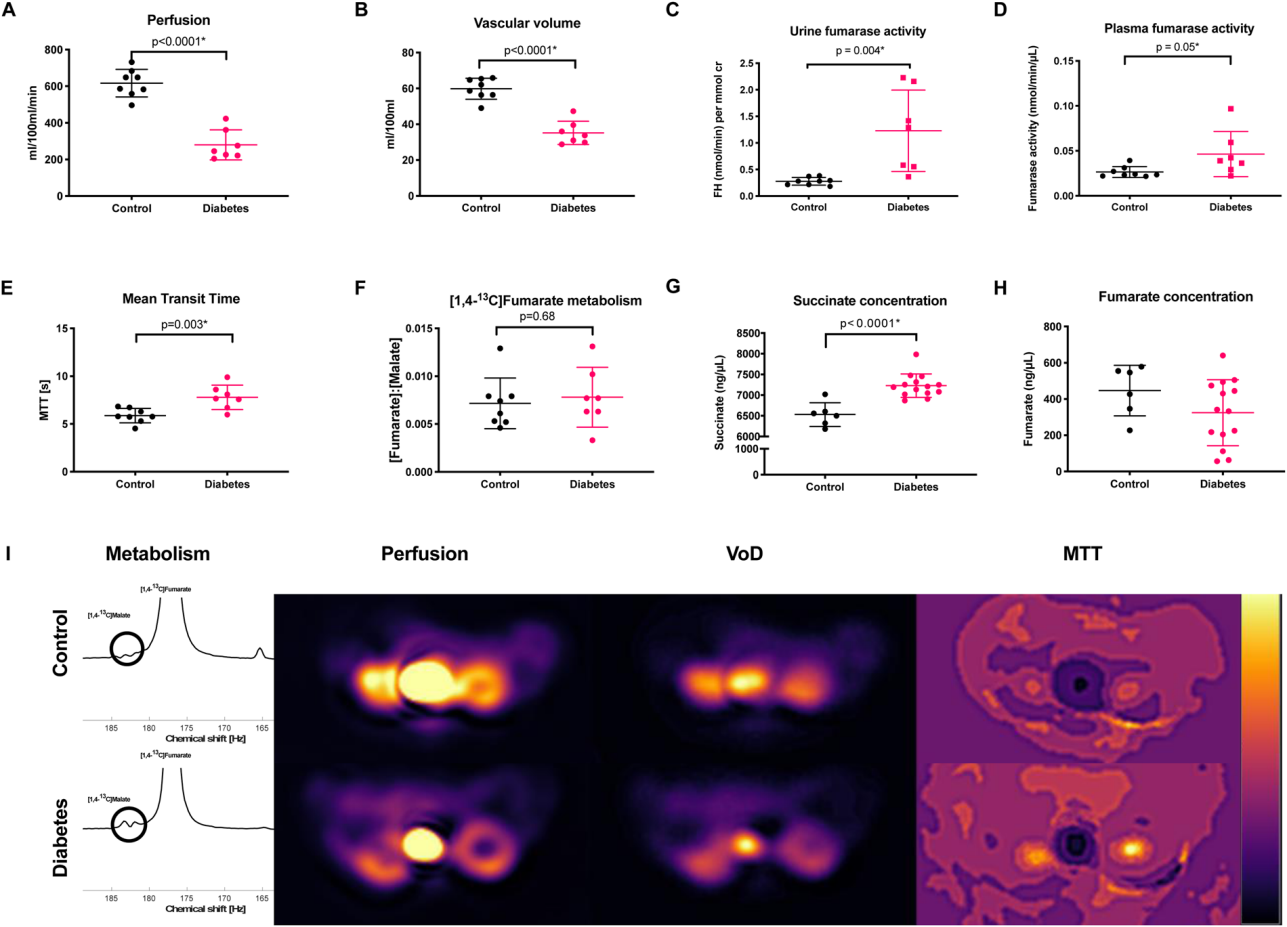
(**A,B**) A significant lower [1,4-^13^C_2_]fumarate perfusion and volume of distribution (VoD) was seen in diabetic rats compared to controls. (**C,D**) Diabetes was associated with an increased urinary and plasma FH activity after 14 days of diabetes. (**E**) A significant longer [1,4-^13^C_2_]fumarate mean transit time (MTT) was found in diabetic rats compared to controls. (**F**) A similar [1,4-^13^C_2_]fumarate-to-[1,4-^13^C_2_]malate conversion was seen between the kidneys of diabetic rats and control rats 14 days after induction of diabetes. (**G)** Diabetes was associated with an increased renal tissue succinate concentration. (**H)** No difference was found in renal tissue fumarate levels. (**I**) Top and bottom row show a control and diabetic rat respectively, with a spectrum showing the fumarate (central peak) and malate peaks (Highlighted by a bold circle), perfusion, volume of distribution, and mean transit time maps.

The ^13^C/^1^H images were converted to the DICOM format and analyzed using Osirix software^32^. The [1,4-^13^C_2_]fumarate was analyzed with a model free deconvolution in the UMMperfusion tool^33^ and correcting for T1 relaxation as previously described^34,35^, resulting in a perfusion, volume of distribution and a mean transit time of the [1,4-^13^C_2_]fumarate signal. A ratio-metric analysis of the conversion of [1,4-^13^C_2_]fumarate to [1,4-^13^C_2_]malate was performed by area under the curve (AUC), on the spectroscopic data due to the low malate level compared to previous reported values in an AKI model^8^. Fitting was performed using an in-house MATLAB general linear model fit of the spectroscopic data (See time curve in appendix). A representative image of [1,4-^13^C_2_]fumarate and [1,4-^13^C_2_]malate were overlaidon anatomical ^1^H images (Fig. 3I).

### Sample preparation for label-free protein quantification by LC-MS/MS

Protein concentration was determined by BCA assay, and 40 µg protein per sample (cell line NRK-52E with and without glucose as above) was separated by SDS-PAGE (Any kD, 26 wells, Bio-Rad) for 30 min at 200 V, and stained with Coomassie Brilliant blue followed by destaining. Each sample lane was cut into three gel pieces, and proteins were reduced, alkylated, in-gel trypsin digested, and peptides purified as previously described^36^.

### LC-MS/MS analysis

Peptide samples were trapped and separated by liquid chromatography (LC) (Easy-nLC 1000, Thermo Scientific) using pre-column (EASY column, C18, 5 µm, 120 Å, 100 µm x 2 cm, Thermo Scientific) and analytical column (EASY column, C18, 3 µm, 120 Å, 75 µm x 10 cm, Thermo Scientific) in a 90 minute gradient (4–36% acetonitrile) in 0.1% formic acid, online coupled to mass spectrometry (MS). A Q-Exactive Plus mass spectrometer (Thermo Scientific, Bremen) operating in positive mode using higher energy collisional dissociation (HCD) with a normalized energy (NCE) of 30 was used to acquire mass spectra. Mass resolution in full scan (MS1) was set to 70,000 with scan range between 392–1600 m/z. Up to the 10 most intense peaks in MS1 were fragmented in MS2 using data dependent acquisition. MS2 resolution was set at 17,500. Unassigned and +1 charge state molecules were excluded from fragmentation, and a dynamic exclusion of 15 seconds was used.

### Proteomics data treatment

Protein identification and quantification was performed using MaxQuant (version 1.5.3.30)^37^. Raw files were searched against the rat sequence database (fasta file of *Rattus norvegicus* from UniProt, 8,079 reviewed sequences, 09/24/2019). Oxidation of methionine was set as dynamic modification and cysteine carbamidomethylation as static modification. Protein and peptide false discovery rates (FDR) were set to 0.01. Furthermore, match between runs and iBAQ protein intensity values were used. Variation between samples was accounted for by normalizing the iBAQ intensities to the mean iBAQ intensity of each sample. Only proteins with two unique peptides were included in the statistical analyses, and only proteins with quantitative data in at least six out of the eight samples were included. When iBAQ was zero, randomized values under the 2% quartile were imputed. For proteins found in more than one gel fraction, the iBAQ values were summed.

Differentially regulated proteins were filtered out based on two quantitative criteria; fold change (FC) and p-value from t-test. FC was calculated as mean of the treated group divided by the mean of the untreated group. A two-way t-test assuming equal variance was performed on log-transformed iBAQ intensity data. Functional annotation cluster analysis was performed on the significantly regulated proteins using the online available DAVID Bioinformatics Resources 6.8.

### Statistics

All data are presented as means ± SD. Normality was assessed with quantile plots. A P-value <0.05 was considered statistically significant. A two-way repeated ANOVA was used to investigate the functional parameters over time between the control and diabetes group.

## Results

In order to investigate the early alterations in enzyme and metabolite leakage in the diabetic kidney, diabetic (n = 7) and control rats (n = 8) were placed in metabolic cages for 24 hours at 2 and 7 days after induction of diabetes and scanned 14 days after induction of diabetes. Immediately hereafter, blood and urine samples were withdrawn and the kidneys were excised, and FH activity was measured in the samples (Fig. 2E,G). Healthy controls had a generally higher body weight compared to diabetic rats as early as two days following the STZ induced diabetes (Fig. 2H). Diabetic rats had an increased blood glucose level (Fig. 2I), urinary output (Fig. 2C), and venous FH activity (Fig. 2E) following induction of diabetes, as well as lower plasma (Fig. 2B) and urinary creatinine (Fig. 2F) (Cr) levels. However, no change was observed in the creatinine clearance (Fig. 2D) and the urine FH activity (Fig. 2G) during the first seven days, when normalizing to urinary creatinine concentration. Two weeks of untreated diabetes resulted in renal hypertrophy with a statistically significant increased kidney weight (*p* = 0.0004) in diabetic rats (1.77 ± 0.08 g, n = 6) compared to controls (1.13 ± 0.10 g, n = 8).

### Vascular complications and renal functional decline after 14 days of diabetes

Fourteen days after induction of diabetes, there was a statistically significant reduction in [1,4-^13^C_2_]fumarate perfusion down to 55% (*p* < 0.0001) (Fig. 3A) and volume of distribution (VOD) 41% (*p* < 0.0001) (Fig. 3B) concomitant with an increased mean transit time (MTT) of 33% (*p* = 0.0032) (Fig. 3E) when comparing the diabetic animals to the controls. The [1,4-^13^C]fumarate T_1_ was estimated from the *in vivo* T_1_ of the control relaxation rates, assuming no metabolic conversion. The T_1_ was found to be 16±1 s.

Fourteen days of diabetes manifested in an increased urininary (*p* = 0.004) and plasma (*p* = 0.05) FH activity (Fig. 3C,D, respectively), while no difference in the [1,4-^13^C_2_]malate production was seen in the diabetic kidney compared with healthy controls (Fig. 3F). A significantly 11% increased succinate pool was observed in the diabetic kidneys compared to normoglycemic controls (*p* < 0.0001) (Fig. 3G), while no difference was seen in the fumarate pool size (*p* = 0.16) (Fig. 3H). Representative spectra as well as maps of VOD, MTT and perfusion are illustrated (Fig. 3I).

### Fumarate hydratase leakage is linked to renal function

It is well-known that perfusion is directly linked to glomerular filtration rate, the so called tubuloglomerular feedback^38^, and we thus investigated the correlations between plasma creatinine and the various FH-linked measurements. A significant positive correlation was observed between plasma creatinine levels and 1) the renal fumarate perfusion (R^2^ = 0.39, *p* = 0.02) and 2) the volume distribution (R^2^ = 0.44, *p* = 0.01), while there was no such correlation between the mean transit time and the plasma creatinine (R^2^ = 0.13, *p* = 0.21) or the hyperpolarized renal fumarate-to-malate conversion (R^2^ = 0.01, *p* = 0.74) and the plasma creatinine. On the otherhand the systemic measure of cell death via the plasma FH activity (R^2^ = 0.36, *p* = 0.02), and the proteinuria related urinary FH activity (R^2^ = 0.45, *p* = 0.01) were negatively correlated with the plasma creatinine.

### Cellular integrity and sensitivity – Hyperglycimia promotes SDH dissociation

**Figure 4:**
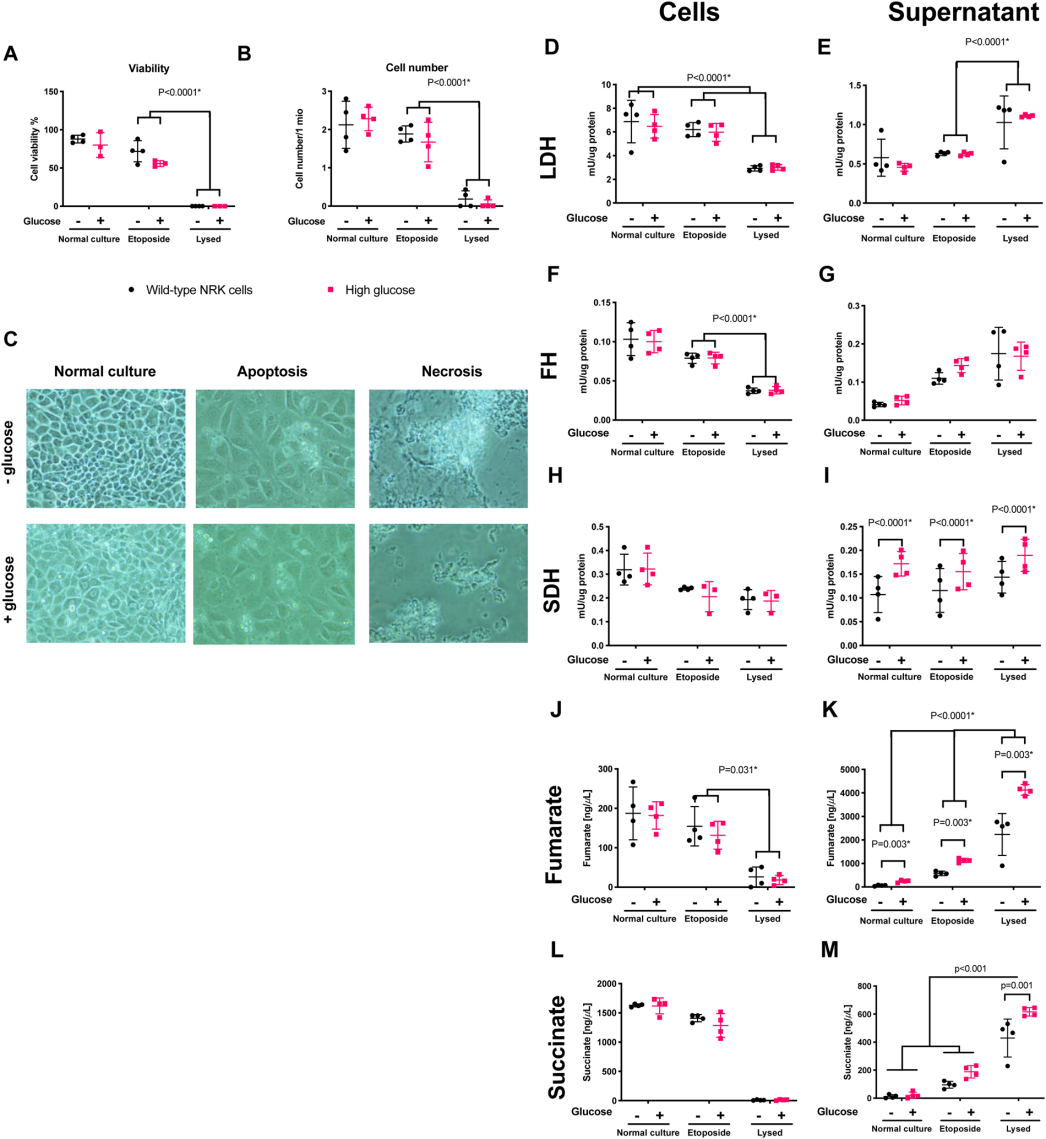
(**A,B**) Cell viability and cell number after exposure, showing a decreasing viability and cell number with etoposide treatment promoting an apoptotic phenotype (slight numerical decreased cell number of 19% although it did not reach statistical significance (*p* = 0.11) and 20% decreased viability (*p* = 0.002)) and lysed cells promoting a necrosis phenotype. **C**. Microscopic images of the cells following exposure. Magnification in the images were 40×. (**D-I**) LDH, FH and SDH activity in cell and supernatant fractions, showing similar inverse response to the two cell death inductions for LDH and FH. SDH on the other hand did not demonstrate this behavior, being largely unaffected in cell activity, while the extracellular fraction was increased in hyperglycemic cells. (**J-M**) The metabolic concentrations of fumarate (cell death, *p* < 0.0001 and glucose, *p* < 0.5) and succinate (cell death, *p* < 0.0001 and glucose, *p* = 0.3) in cells was highly dependent on cell death. The supernatant fractions was highly dependent on both cell death and hyperglycemia, showing an increased exctracllular accumulation of fumarate (cell death, *p* < 0.0001 and glucose, *p* < 0.0001) and succinate (cell death, *p* < 0.0001 and glucose, *p* = 0.0012).

In order to verify the origin of the increase in urinary and blood FH activity, a series of cell culturing experiments were performed with renal tubular cells with and without high glucose (25 mM) in the culturing medium. A statistically significant effect of both glucose culturing (*p* = 0.003), apoptosis (15 μM etoposide for 24 hours) and necrosis (3 cycles of freeze and thaw) (*p* < 0.0001) was seen on the fumarate levels in the supernatant (Fig. 4K), while only apoptosis and necrosis showed increased levels of fumarate intracellularly (*p* = 0.031) (Fig. 4J). LDH activity was only elevated by apoptosis and necrosis extracellularly (*p* < 0.0001) (Fig. 4E), and no such effects were seen intracellularly (*p* = 0.479) (Fig. 4D).

The enzyme activities were largely similar between glucose and normal culture (Fig. 4D–I), while a clear dependency on cellular integrity was seen (Fig. 4A–C). A more pronounced differentiation was seen with the individual metabolites with significant differences between etoposide (apoptosis) and lysed cells (necrosis) for both fumarate and succinate (Fig. 4J–M). This indicates 1). That the effects of hyperglycemia and apoptosis are additive with regard to releasing metabolites 2). That the kidney fumarate-to-malate conversion mainly is driven by apoptosis/necrosis loss of enzyme and metabolites under early diabetes conditions.

A generally increased SDH activity was seen in the supernatant for all glucose cultured cells (*p* = 0.003) (Fig. 4I).

### Protein regulation – Hyperglycemia promotes SDH regulation and uncoupling of the electron transport chain

To further delineate intracellular effects of elevated glucose concentrations in cultivated renal tubular cells an exploratory mass spectrometry (MS) based proteomics study was applied. A total of 1,549 proteins were identified and quantified in the cell culture samples. Of the total identified proteins, 171 were significantly regulated by hyperglycemic conditions, see Table S2. Hyperglycemia was associated with a significant and pronounced reduction of the glucose transporter, GLUT1 (FC = 0.35).

**Figure 5:**
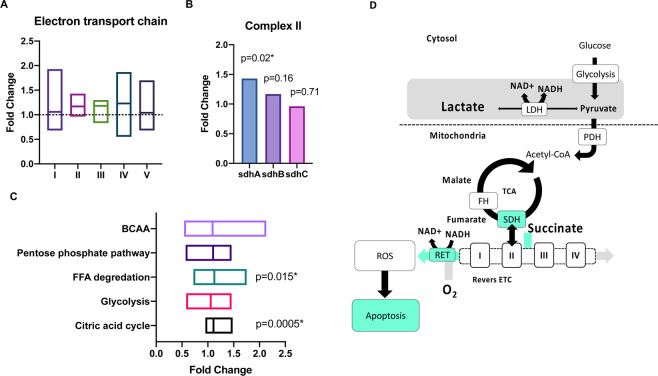
(**A**) Proteomics representation showing the electron transport chain (ETC) protein regulation (**A**) with a tendency towards an upregulated complex II in contrast to the rest of the complexes. (**B)** SDH-A was significantly upregulated, while SDH-B and SDH-C was not, indicating a mismatch between the TCA cycle enzyme and the rest of the complex. (**C**) A general upregulation of FFA degradation and TCA cycle proteins was found if testing the mean of the individual distributional patterns was different from 1 using a single sample t-test. (**D**) Hypothesized mechanisms for the HIF independent pseudo-hypoxic phenotype; Hyperglycemia induced increased glycolytic activity, ultimately increasing the intracellular lactate pool, changing the redox potential and lowering intracellular pH, while at the same time maintaining the PDH flux^11,15,16,20^. This leads to an SDH subunit dissociation (increased SDH-A levels compared to SDH-B), which uncouples the TCA and ETC^39^, thus reversing the ETC thereby leading to pronounced leak respiration^25^ and accumulating mitochondrial ROS production^40^, which triggers a HIF independent apoptosis reaction.

A significant upregulated antioxidant capacity was found in the hyperglycemic cells with four significantly regulated proteins; Peroxiredoxin-1, Peroxiredoxin-4, Glutathione synthetase, and mitochondrial superoxide dismutase. SDH-A was significantly upregulated, while SDH-B and SDH-C were not (Fig. 5A,B). Two glycolytic proteins, triosephosphate isomerase 1 and phosphofructokinase, were significantly upregulated under hyperglycemic conditions. TCA cycle proteins were generally upregulated; 17 of 20 had a FC > 1 and of those, two were significant (Fig. 5C). Four proteins of fatty acid degradation pathway were significantly upregulated (Fig. 5C). No general tendency was observed in degradation of branched chain amino acids (BCAA) (Fig. 5C) but three proteins were significantly upregulated. Using functional annotation clustering, three functional clusters were identified; Cluster 1 containing proteins annotated as lysosomes and glycosylation-related proteins (15–32 proteins), Cluster 2 containing proteins annotated as glycosidases and carbohydrate metabolic processes (7–8 proteins), and Cluster 3 containing proteins annotated to lipid metabolism (14 proteins).

## Discussion

The main findings of this study are the increased and maintained FH blood pool activity (systemic cell death) as early as 48 hours after induction of diabetes and that the hemodynamic and urinary FH activity changes (proteinuria) in the diabetic rats two weeks after induction of diabetes, while no kidney specific acute necrosis was observed.

Cell death is associated with an increased level of extracellular FH enzymes, requiring no co-substrates to convert fumarate to malate and thus drives a cell independent enzymatic conversion in situations of cell death. The comparable renal [1,4-^13^C]malate production in the diabetic kidneys compared to controls 14 days after induction of diabetes supports apoptosis as the main cell death pathway. This is supported by the fact that apoptosis in general is less intense in hyperpolarized [1,4-^13^C]malate signal than necrotic renal damage^8,21^.

The hemodynamic alterations, i.e. change in volume of distribution is likely originating from early renal microvascular complications, increased proximal reabsorption and/or hyperfiltration associated with diabetes. This change is typically not seen with the larger conventional gadolinium complex tracers this early in the development of diabetes^7^ (see supplemental Fig. 2). This supports microvascular complications as an increase in the mean transit time could result from either hypertropy increased leakage of [1,4-^13^C]fumarate to the interstitium without FH enzyme present or loss of FH enzyme to the urine. The lower plasma creatinine levels and the maintained FH urine activity support early hyperfiltration and increased proximal reabsorption of small molecules and no loss of FH to the urine. These findings are consistent with previous reports of altered hemodynamic properties associated with vascular leakage in cancers, showing a dependent metabolic conversion of the substrate delivery^41^. This indicates that microvascular complications associated with diabetes can be seen earlier with hyperpolarized ^13^C MRI than with conventional MRI and thus shows great promise for future applications in various diseases.

Interestingly, only SDH showed glucose culturing sensitivity, indicating SDH as a key regulator in the diabetic phenotype. The membrane bound SDH complex links the electron transport chain (ETC) complex II and the TCA cycle and changes in intracellular pH has been shown to introduce SDH subunit dissociation. This dissociation uncouples the TCA cycle and ETC, which promote oxidative stress and subsequent apoptosis^39^. Thus we speculate, that SDH could be responsible for the diabetic pseudo-hypoxic condition^39^, by uncoupling the oxygen utilization and the TCA cycle (increased lactate production and lowering of intra-cellular pH)^11,15,16,19,20,42^.

Of note, the proteomics results showed perturbed stoichiometry of the measured SDH-subunits, with an increased SDH-A protein concentration, compared to both SDH-B, SDH-C and the complete ETC. We speculate that this is a reflection of dissociation of SDH complex, and uncoupling of the TCA cycle from the ETC flux, which could be a defining moment in the development of hyperglycemia induced renal reprogramming. This reprogramming is likely reversing ETC complex II by the increased pool of succinate and thus directly increasing the ROS production^40^ (Fig. 5D). This is supported by the fact that fumarate levels were not changed upstream of SDH and more importantly suggest fumarate accumulation and subsequent fumarate-dependent regulation of HIF as a key difference between the phenotypes seen in renal cancer and in early diabetes^24,43^.

The TCA cycle flux was most likely maintained by increasing the TCA cycle enzymes as seen by the proteomics results. This could indicate that this mechanism for decoupling and reversing the ETC under hyperglycemic conditions is a protective mechanism designed to trigger programmed cell death and apoptosis. This is supported by the functional clustering analysis demonstrating a significant upregulation of proteins regulating lysosomes as well as post translational modifications by glycosylation and glycoxidation, which plays important roles in apoptosis.

Elevated levels of cytosolic fumarate and succinate concentrations have previously been shown to promote stabilization of HIF-α, and thus promote a hypoxic response in diabetic mouse kidneys^44^. Investigations of the oxygen dependent pathways indicate that the HIF related genes HO-1 and VEGF were not yet activated, although an oxygen sensitivity has been observed with antioxidant treatment, showing a reduction in the FH activity^20^. The mechanism for this could be an increased apoptotic phenotype, as antioxidant treatment protects against apoptosis, and lack of anti-oxidant genes increases apoptosis in response to oxidative stress. Our results suggest an upregulation of antioxidant proteins and as such a potential preventive mechanism to protect against the SDH promoted reverse electron transport (RET) stress and subsequent apoptosis (Fig. 4D). This mechanism deviates from the HIF activated Warburg effect seen in many cancer phenotypes^43,45–47^, where a causal link between inactivation of FH and/or loss of FH enzyme leads to accumulation of succinate and fumarate, which subsequently leaves the mitochondria and inhibits prolyl hydroxylase in the cytosol, thereby inhibiting the HIF1-α hydroxylation^43^. Interestingly, FH inactivation has been associated with non-malignant cyst growth with an overexpression of HIF1-α and related genes^47^. We have previously shown a decreased fumarate concentration in kidney cortex in diabetes and further decrease following antioxidant treatment, whereas an increased succinate concentration was largely independent of antioxidant treatment^20^. The *in vivo* and cell data seem to suggest that enzymes and metabolites are lost to the extracellular compartments and is not accumulated in the cytosol, allowing inhibition of HIF1-α early in the diabetic kidney. It is currently unknown why the HIF stabilization, associated with reduction of key TCA cycle enzymes such as FH and SDH and the metabolic shift towards glycolysis^43^, results in advantage to diabetic cells^25^, and why the diabetic rats at least in early diabetes is lacking this activation. We have previously shown an increased HIF stabilization following succinate buildup in acute ischemic injury^18^ as well as a significant increased renal and biofluid FH activity^8^. Our data show an upregulation of antioxidant proteins concomitant with the upregulated glycolysis and TCA cycle proteins, which could potentially be an earlier event.

A better differentiation of the underlying populations of cell death could have been investigated by H&E, IHC, caspace-3 staining and or flow cytometry analysis of Annexin-V/PI staining and as such further studies are needed to fully understand the mechanisms associated with hyperglycmia mediated renal cell death.

In conclusion, we have shown a remarkable change in the hemodynamic response of fumarate in the diabetic kidney which is likely originating from hypertrophic and microvascular changes as well as early hyperfiltration and proximal reabsorption. The enzyme loss seen in early diabetes is dominated by an apoptotic response (HIF independent pseudo-hypoxia), which is likely originating directly from the metabolic reprogramming associated with hyperglycemia and thus linking the glycolysis, mitochondrial uncoupling, and the TCA cycle with the features seen in the more well-known Warburg metabolic phenotype^44^. It is currently unknown if the two phenotypes co-exist in a sequential manner, or if there are two very distinct phenotypes. Both call for better methods for stratifying the phenotypes, and this supports the use of hyperpolarized MR as a method allowing differentiation of the two phenotypes by using hyperpolarized [1-^13^C]pyruvate or [2-^13^C]pyruvate since the entrance to the TCA cycle seem to be a defining point in the distinction of the two. The use of hyperpolarized [1,4-^13^C]fumarate enables detailed characterization of the underlying cell death mechanisms as well as potential earlier signs of vascular complications. Taken together this and previous studies support further exploration of metabolic imaging in particular hyperpolarized MR in human subjects for determining the value in clinical renal disease research questions.

## Supplementary information

Supplementary information.

Supplementary information1.
