# Supplementary material for: Hyperpolarized [1,4-13C]fumarate imaging detects microvascular complications and hypoxia mediated cell death in diabetic nephropathy
Source: Sci Rep. 2020 Jun 15;10:9650. doi: 10.1038/s41598-020-66265-6 (PMC7295762; doi:10.1038/s41598-020-66265-6)
Supplement: Supplementary file 2 — Supplementary information1. [file 41598_2020_66265_MOESM2_ESM.docx]

**Hyperpolarized [1,4-^13^C]fumarate imaging detects microvascular complications and hypoxia mediated cell death in diabetic nephropathy**

Christoffer Laustsen PhD^1^, Per Mose Nielsen Msc^1^, Haiyun Qi MD^1^, Mette Hadberg Løbner Msc^2^, Johan Palmfeldt PhD^2^, Lotte Bonde Bertelsen PhD^1^

^1^MR Research Centre, Department of Clinical Medicine, Aarhus University, Aarhus, Denmark

^2^Research Unit for Molecular Medicine, Department of Clinical Medicine, Aarhus University, Aarhus, Denmark

**mRNA gene expression does not support HIF activation**

The functional and metabolic changes in kidney tissue were not complemented by an increased expression of fumarate hydratase, FH1 (p=0.65) and FH2 (p=0.26), or SDH A (p=0.94) nor by an increased expression of HIF related proteins, erythropoietin (EPO) (p=0.31), Heme oxygenase 1 (HO-1) (p=0.13), vascular endothelial growth factor (VEGF) (p=0.79) (Figure S1). Used primers are shown in table S1

**Figure S1. qPCR mRNA expression**

**Figure S2. Comparison of Perfusion tracers in diabetes and control rats**

To confirm the lower perfusion using hyperpolarized [1,4-^13^C]fumarate an additional comparison to 1H DCE and [1-^13^C]pyruvate perfusion was investigate using previous acquired data. The hemodynamic properties of the hyperpolarized tracers [1-^13^C]pyruvate (p<0.0001) and [1,4-^13^C]fumarate (p<0.0001) are different in diabetic kidneys compared to controls as well as using a conventional gadolinium contrast agent dynamic contrast enhanced (DCE) perfusion.

**Figure S3. Top: Fumarate and Malate images from a rat showing the low SNR malate and the high SNR fumarate images in the kidneys. Lower: Temporal dynamics of the spectral information of the hyperpolarized [1,4-^13^C]fumarate experiment**

The mean of all rat time curves confirms the low production of malate on the timescale of the experiment.

**Table S1. Primer sequences**

| Gene | Forward Primer Sequence | Reverse Primer Sequence |
| --- | --- | --- |
| 18s | 5’-CAT GGC CGT TCT TAG TTG-3’ | 5’-CAT GCC AGA GTC TCG TTC-3’ |
| FH1 | 5’-CTGAAGGTCCCAACCGATAA-3’ | 5’-CATCTGCGGCCTTCATTATT-3’ |
| FH2 | 5’-TGAAGGTCCCAACCGATAAG-3’ | 5’-CATCTGCGGCCTTCATTATT- 3’ |
| SDHA | 5’-CAGGACTGAAGATGGGAGGA-3’ | 5’-ACCGCAGAGATCGTCCATAC-3’ |
| VEGF | 5’-CAATGATGAAGCCCTGGAGT-3’ | 5’-TTTCTTGCGCTTTCGTTTTT-3’ |
| HO-1 | 5’-TCT ATC GTG CTC GCA TGA AC-3’ | 5’-AAG GCG GTC TTA GCC TCT TC-3’ |
| EPO | 5’-AGTCGCGTTCTGGAGAGGTA-3’ | 5’-AGGATGGCTTCTGAGAGCAG-3’ |

**Proteomics.**

**Supplemental table S2.** Functional annotation cluster analysis of the significantly regulated proteins was performed in DAVID Bioinformatics Resources 6.8 using default settings. The 171 proteins significantly regulated by hyperglycemic conditions, based on p-value and fold change (FC) (p < 0.05 and 1/1.15 > FC > 1.15), were analyzed with the altogether detected 1,549 proteins as a background. Clusters with enrichment scores above 1.3 (p<0.05) are shown. Count is the number of proteins in the cluster, FDR=false discovery rate.
